# Novel biologically active polyurea derivatives and its TiO_2_-doped nanocomposites

**DOI:** 10.1080/15685551.2020.1767490

**Published:** 2020-05-25

**Authors:** Mahmoud A. Hussein, Khalid A. Alamry, Samar J Almehmadi, M.A. Elfaky, H. Džudžević-Čančar, Abdullah M. Asiri, Mostafa A. Hussien

**Affiliations:** a Department of Chemistry, Faculty of Science, King Abdulaziz University, Jeddah, Saudi Arabia; b Polymer Chemistry Lab., Chemistry Department, Faculty of Science, Assiut University, Assiut, Egypt; c Faculty of Pharmacy, Natural Products and Alternative Medicine Department, King Abdulaziz University, Jeddah, Saudi Arabia; d Department of Natural Science in Pharmacy, Faculty of Pharmacy, University of Sarajevo, Sarajevo, Bosnia-Herzegovina; e Center of Excellence for Advanced Materials Research, King Abdulaziz University, Jeddah, Saudi Arabia; f Department of Chemistry, Faculty of Science, Port Said University, Port Said, Egypt

**Keywords:** Polyurea derivatives, nanocomposites, TiO_2_, antibacterial activity, molecular docking

## Abstract

A new series of polyurea derivatives and its nanocomposites were synthesised by the solution polycondensation method through the interaction between 4(2-aminothiazol-4-ylbenzylidene)-4-(tert-butyl) cyclohexanone and diisocyanate compound in pyridine. The PU_1–3_ structure was conﬁrmed using Fourier transform-infrared (FTIR) spectroscopy and characterised by solubility, viscometry, gel permeation chromatography (GPC), and X-ray diffraction (XRD) analysis. In addition, PU_1–3_ was evaluated by TGA. Polyurea–TiO_2_nanocomposites were synthesised using the same technique as that of PU_1–3_ by adding TiO_2_ as a nanofiller. The thermal properties of PU_2_TiO_2_a–d were evaluated by TGA. Moreover, the morphological properties of a selected sample were examined by SEM and TEM. In addition, PU_1–3_ and PU_2_TiO_2_a–d were examined for antimicrobial activity against certain bacteria and fungi. The PU_1–3_ showed antibacterial activity against some of the tested bacteria and fungi, as did PU_2_TiO_2_a–d, which increased with the increase in TiO_2_ content. Furthermore, molecular docking studies were displayed against all PU_1–3_ derivatives against two types of proteins. The results show that the increase in the strength of π–H interactions and H-donors contributed to improved binding of PU2 compared to PU1 andPU_3._ The docking of 1KZN against the tested polymers suggests an increase in the docking score of PU_2,_ then PU_1_, and PU_3_, which is in agreement with the antibacterial study.

## 1. Introduction

Polyurea derivatives are important polymers obtained by the interaction between a diisocyanate and an amine. Polyurea is an extremely rough material and exhibits high hardness and good chemical resistance, which enable it to have many applications, for example, in corrosion protection and coating systems [1]. Many efforts to synthesise a new class of thermally stable polyurea to achieve different properties have been reported, such as phosphorus-containing heterocyclic polyurea [2–5]. Heterocyclic-based PU derivatives or by another wards, metal-containing PU with an ionic link in the main chain was synthesized and received great attention [6,7]; meanwhile, the introduction of a metal into heterocyclic polyurea, to achieve a versatile number of variable properties, has been studied, and the polymer chain was reported as a possible cause for their increased thermal stability [2,3,5,8].

Several techniques have been used to synthesise polyurea, but the most effective technique is to react diamine with diisocyanate. This reaction is a step-growth addition reaction of amine across the carbon-nitrogen double bond, and there is no by-product. The essential components for a compound to be an efficient inhibitor are [9]: (i) it should be chemisorbed onto the metal surface, (ii), it should form a defect-free compact barrier ﬁlm, (iii) it should be polymeric or polymerise in situ on the metal, and (iv) the barrier thus formed should increase the inner layer thickness. Compounds containing nitrogen, sulfur, and oxygen have been established as good inhibitors for iron in acidic media [9]. The *π* bonds inorganic compounds are found in corrosion inhibition of steel by being adsorbed through electron sharing on the electrode surface [10]. The presence of functional groups, for example, NH,N = N, CHO, and RــOH, in the inhibitor molecule [11], steric factors, aromaticity, and electron density of the donor atoms are found to inﬂuence the adsorption of the inhibitor molecule over the corroding electrode surface.

Recently, some polymers [12–14] have received intensive attention because of their wide application range. This work continues our previous studies regarding the preparation of various types of organic polymers and/or polymer nanocomposite materials having interesting properties and thus can find widespread applications in different fields of study [15–31].

According to our best of knowledge, there are no previous reports for PU derivatives reinforced by TiO_2_ nanoparticles in the form of nanocomposites. A general understanding which describes the role of reinforced inorganic material throughout the nanocomposite formation and about how organic polymer interacts with inorganic particles in the nanoscale to form a composite material has been reported in the literature [32–36]. In this study, we address the synthesis and characterization of new polyurea derivatives containing diarylidenecyclohexanon moiety in the polymer and prepare the polymer nanocomposite using TiO_2_ nanoparticles. TiO_2_ is a commonly used material because it is inexpensive, is non-toxic, has a high refractive index, can be used as a broadband UV filter, is chemically inert, exhibits biological activity against bacteria via photo-irradiation, is corrosion-resistant, and has high rigidity [37].

The main goal of the present work is to synthesize a new series of polyurea derivatives and its related TiO_2_nanocomposites. The prepared materials are characterized by investigating their crystallinity, thermal stability, solubility, viscometry, morphology. Moreover, the GPC measurement is also used to determine the molecular weight of the synthesised polyurea. Finally, investigate their antimicrobial properties as well as molecular docking studies.

## 2. Experimental method

### 2.1. Measurements

IR spectra were recorded on an IR-470, infrared spectrophotometer. The ^1^ HNMR spectra were obtained on GNM-LA (400 MHz) spectrophotometer at room temperature in CDCl_3_ using TMS as a reference. Inherent viscosities of polyurea solutions were measured in dimethyl sulfoxide (DMSO) at 30°C using an Ubbelohde suspended-level viscometer. The polymer solubility was tested for powdery samples at room temperature under the same conditions with different solvents: formic acid, chloroform (CHCl_3_), dichloromethane (CH_2_Cl_2_), benzene, dimethylformamide (DMF), sulfuric acid, and DMSO. The molecular weight of polyurea was evaluated by GPC using an Agilent-GPC from Agilent Technologies in Germany. The refractive index detector was G-1362A with 100–104–105 A° Altrastyragel columns connected in series. The eluent used was DMF at a flow rate of 1 mL/min and in the presence of PS as a reference polymer. The GPC apparatus was run under the following conditions: flow rate = 2.000 mL/min, injection volume = 100.000 μL, and sample concentration = 1.000 g/L. X-ray diffraction (XRD) measurements of polyurea derivatives and polyurea–TiO_2_ nanocomposites with different TiO_2_ nanoparticle contents (2%, 5%, 10%, and 20%) were recorded on an XRD D8 Discovery model manufactured by the Bruker Company in Germany. XRD measurements were conducted at 40 kV and 40 mA over a scanning range from 5° to 90°at a scan speed/duration time of 4.0000 deg/min. Thermal analysis was performed by thermogravimetric analysis (TGA) using the Shimadzu-D50 thermal analyser model manufactured by the Shimadzu Company (Japan). The rate of temperature increase was 10°/min, holding temperature 800°Cusing platinum cell and air atmosphere. Transmission electronic microscopy (TEM) measurements were recorded using an EM-2100 High-Resolution model at 25X magnification and 200 kV. TEM images were taken only for polyurea–TiO_2_ nanocomposites with 20% TiO_2_ nanoparticles. SEM images were taken on a Jol 2000 (Japan) model for polyurea–TiO_2_ nanocomposites with 2% and 20% TiO_2_ nanoparticles.

### 2.2. Reagents and solvents

TiO_2_nanoparticles with a size of <50 nm and 4-tert-butylcyclohexanone (Merck, Germany) were used as purchased. Chloroacetyl chloride and thiourea (Merck, Germany) were used as received. Anhydrous aluminium chloride (Aldrich, Germany) was used as purchased, while hexamethylene diisocyanate, 4,4ʹ-diphenylmethane diisocyanate, 1,4-phenylenediisocyanate (all from Aldrich, 97%), and pyridine (Merck, Germany) were dried using sodium hydroxide pellets for three days. Methanol (absolute, 99.8%), ethanol (absolute, 99.9%), and acetone (Merck, Germany) were used as purchased. Carbon disulfide (Merck, Germany) was dried overnight by calcium chloride, and then distilled under reduced pressure.

### 2.3. Monomer synthesis

#### 2.3.1. 2,6-bis-(benzylidene)-4-(tert-butyl)cyclohexanone (M1)

**Figure 1.**
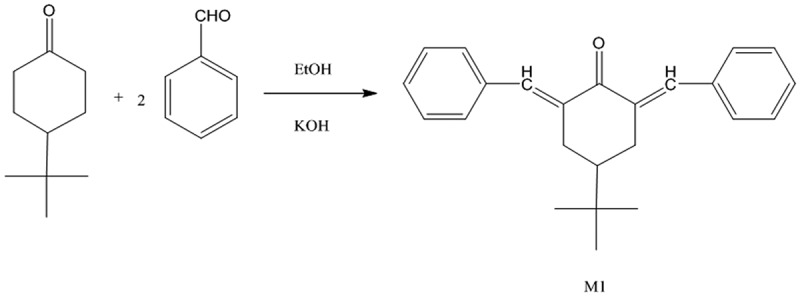
Synthesis of 2,6-bis-(benzylidene)-4-(tert-butyl)cyclohexanone monomer

New monomer, namely, 2,6-bis-(benzylidene)-4-(tert-butyl)cyclohexanone (M1), was synthesised according to the following general procedure. A mixture of 0.02 moles of benzaldehyde and 0.01 mole of 4-(tert-butyl) cyclohexanone in warm ethanol (10 mL of ethanol per gram of the mixture) was prepared. To this was added 10% potassium hydroxide solution dropwise until a yellow solid precipitate was formed. The precipitate was collected, and the remaining solution was stirred for 3–4 h. The precipitate was collected by ﬁltration and then washed with cold water and recrystallized from ethanol, as shown in Figure 1. The FTIR spectra of the monomer showed the absorption bands at 2959 cm^−1^(CH aliphatic), 1661 cm^−1^(C = O of cyclohexanone), and 1604 cm^−1^(C = C). Additional details are found in Figure S1.^1^ HNMR spectrum of 2,6-dibenzylidene4-tert-butyl-cyclohexanone (CDCl_3_) showed peaks at δ = 1.26 (s, 9 H, 3CH_3_), 2.4–2.62 (m, 1 H, CH), 3.15–3.19 (m, 4 H, 2CH_2_ of cyclohexanone), and 7.25–7.78 (m, 12 H, 2CH = C &10Ar–H). Additional details can be found in Figure S4. ^13^CNMR (CDCl3): δ = 27.29, 32.58, 44.44, 128.84, 128.56, 130.36, 136.06, 136.19, 136.87, 190.65. Additional details can be found in Figure S8.

#### 2.3.2. Bis (4-chloroacetylbenzylidene)-4-(tert-butyl) cyclohexanone (M2)

**Figure 2.**
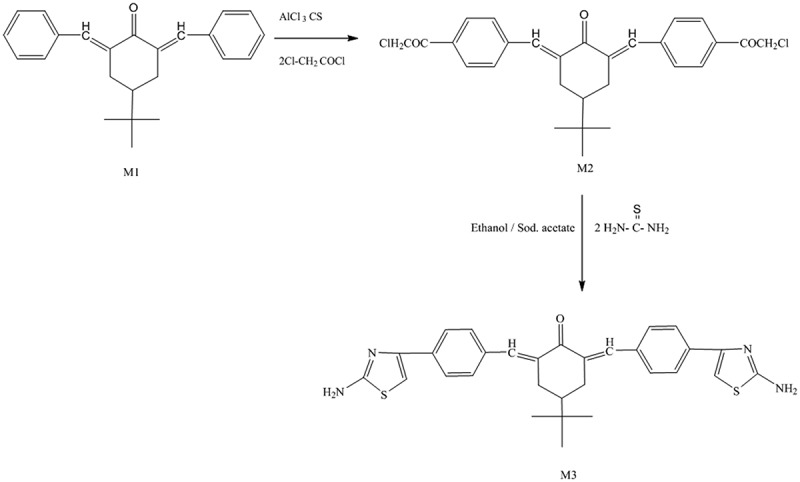
Synthesis of diarylidenecyclohexanone monomers M2 and M3

In a conical flask, 0.02 mole of M1and 0.04 mole of chloroacetyl chloride were added and the mixture dissolved in 80 mL of carbon disulfide. Then, anhydrous aluminium chloride (0.08 mole) was added dropwise. The mixture was stirred in an ice bath for 6 h, then the carbon disulfide was evaporated and the residue poured into cold hydrochloric acid. The yellow-precipitated product was collected by filtration, washed with water, and recrystallized from an appropriate solvent as shown in Figure 2. The IR spectra of this monomer showed absorption bands at 1735 cm^−1^(C = O of chloroacetyl group), with the original absorption bands of 1660 cm^−1^(C = O of cyclohexanone) and 1602 cm^−1^ (C = C) (further information is in Figure S2). ^1^ HNMR spectra (in CDCl_3_) showed peaks at δ = 6.82–7.85 (m, 10 H, 2CH = C & 8 H of aromatic), 4.15 (s, 4 H of CH_2_ chloroacetyl), 3.14–3.18 (m, 4 H, 2CH_2_ of cyclohexanone), 2.41–2.64 (m, 1H, CH), and 0.97 (s, 9 H, 3CH_3_). Additional details are in figure S5. ^13^CNMR (CDCl3): δ 27.29, 29.30, 29.53, 32.58, 44.44, 53.85, 69.53, 128.47, 128.56, 130.31, 136.18, 136.87, 190.66, 210.79. Additional details can be found in Figure S9.

#### 2.3.3. Bis (2-aminothiazol-4-ylbenzylidene)-4-(tert-butyl) cyclohexanone (M3)

A mixture of 0.02 moles of M2 and 0.04 moles of thiourea was dissolved in 40 mL of ethanol and then stirred under reflux for 5 h. The clear solution was poured into cold sodium acetate solution (10%; 25 mL), and the precipitate formed was collected filtered, and re-crystallized using a proper solvent, as shown in Figure 2. The IR spectra showed absorption bands at 3370–3333 cm^−1^, attributed to the primary amino group, and at 1630 cm^−1^(C = N), together with the original bands of the parent monomer at 1660 cm^−1^(C = O of cyclohexanone), 1604 cm^−1^(C = C), and phenylene at 1580 cm^−1^(additional information is in Figure S3). ^1^HNMR spectra (in CDCl_3_) showed peaks at δ = 6.80–7.84 [m, 12 H, 8 H of aromatic and 2 H(CH = C) and 2 H (CH–S)], 5.30 (s, 4 H of NH_2_ exchangeable with D_2_O), 3.18–3.20 (m, 4 H 2CH_2_ of cyclohexanone), 2.47–2.64 (m, 1H, CH), and 0.977 (s,9 H of butyl). More information can be found in Figure S6 and Figure S7 before and after adding the D_2_O respectively. ^13^CNMR (CDCl3): δ 26.90, 27.30, 27.97, 29.34, 29.84, 29.52, 29.68, 29.71, 32.56, 40.95, 44.38, 103.79, 125.99, 128.48, 128.57, 128.59, 130.33, 130.84, 134.71, 135.33, 136.03, 136.07, 136.20, 136.67, 136.84, 136.92, 150.64, 167.60, 190.62. Additional details can be found in Figure S10.

### 2.4. Polymerization process

**Figure 3.**
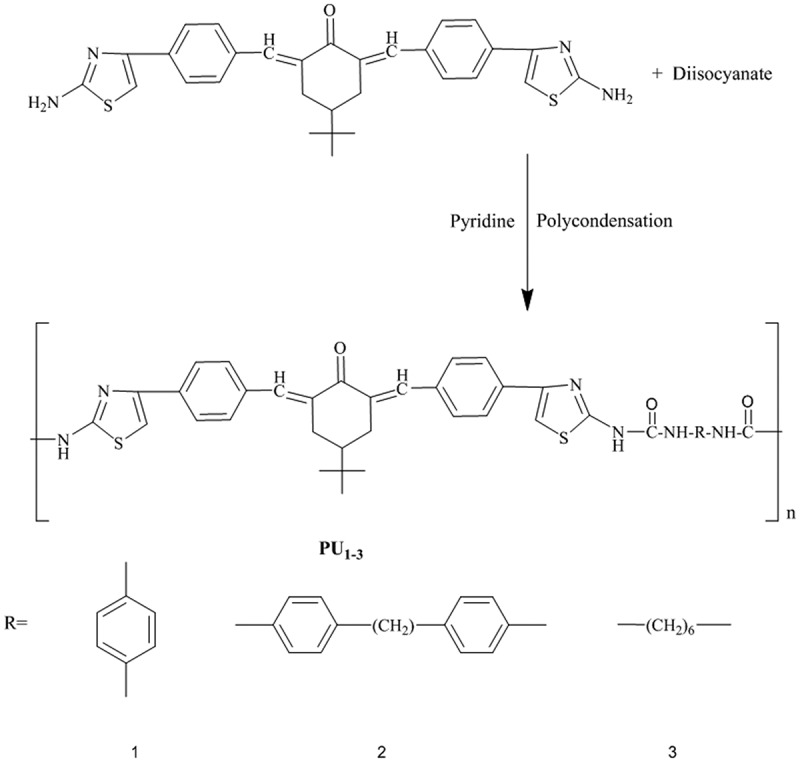
Synthesis of polyurea derivatives PU_1_, PU_2_, and PU_3._

In a three-necked flask equipped with a condenser and dry nitrogen inlet and outlet, a mixture of 0.002 moles of M3 was dissolved in 30–40 mL of dry pyridine. The different aromatic and aliphatic diisocyanates (0.002 moles) dissolved in 15 mL of dry pyridine, were added in a dropwise manner while stirring. After complete addition of the diisocyanate, the reaction mixture was heated under reflux for 15 h and cooled to room temperature. Subsequently, the mixture was poured into ice water, forming a white–brownish precipitate (PU_1_, PU_2_, and PU_3_) as shown in Figure 3. Then, the solid polymers were separated out, filtered, and washed with water. The IR spectra of the polymers showed absorption bands at 3340 cm^−1^(NH of urea derivative) and 1680 cm^−1^ (C = O of urea derivative).

### 2.5. Polyurea-based TiO_2_nanocomposites fabrication process

The PU_2_TiO_2_a–d were synthesised by adding TiO_2_ nanoparticles in different contents (2%, 5%, 10%, 20%) to the solvent (dry pyridine). The mixture was poured into ice water, to remove any excess of pyridine. The same procedure was repeated to synthesise polyurea (see previous section). The polymer data are shown in Table 1. The IR spectra of PU_2_TiO_2_a–d show absorption bands at 3330 cm^−1^(NH of urea derivative) and 1660 cm^−1^(C = O of urea derivative).

**Table 1.** Polymers its related TiO_2_ doped nanocompositesymbols and codes

Code	TiO_2_ nanoparticles (%)	TiO_2_ nanoparticles wt. (g)
PU_1_	0	0
PU_2_	0	0
PU_3_	0	0
PU_2_TiO_2_a	2%	0.036
PU_2_TiO_2_b	5%	0.071
PU_2_TiO_2_c	10%	0.18
PU_2_TiO_2_d	20%	0.36

### 2.6 Antimicrobial activity

Antimicrobial activity was tested for the new synthesised polyurea and polyurea–TiO_2_ nanocomposites by using the agar diffusion method with different bacterial species and fungi: gram-positive bacteria strains (*Bacillus subtilis* and *Staphylococcus aureus*) and gram-negative bacteria strains (*Pseudomonas aeruginosa* and *Escherichia coli*), and fungus (*Candida albicans*). All organisms were kept in the microbiology lab at King Abdulaziz University in Jeddah, Saudi Arabia. The technique used to determine the antimicrobial effect for the new polymer has been described previously [38]. In brief, a 90-mm Petri dish was filled with 25 mL of Muller–Hinton agar; then, 200 μL bacterial cultures were autoclaved for 20 min and were spread on the agar plate surfaces by using sterile swabs. Next, 50 μL of the polymer was added to the agar plates and incubated at 37°C for 24 h. The size of the growth inhibition zone was measured and determined as shown in Table 5.

**Table 2.** Solubility characteristics of PU_1_, PU_2_, and PU_3._

Polymer Code	DMF	HCOOH	CHCl_3_	CH_2_Cl_2_	DMSO	H_2_SO_4_	Benzene
PU_1_	+	+	+	**+ −**	**+**	+	**+ −**
PU_2_	+	+	+	**+ −**	**+**	+	**+ −**
PU_3_	+	+	+	**+ −**	**+**	+	**+ −**

+Soluble at room temperature.

+−Partially soluble.

−Insoluble.

**Table 3.** Inherent viscosities and GPC results for PU_1_, PU_2_, and PU_3._

		GPC data				
Code	M. Formula	^a^Mw	^b^Mn	^c^Pw	DPI	η_inh_(dL/g)
PU_1_	(C_38_H_36_N_6_O_3_S_2_)_n_	38,863.22	35,538.32	~ 56	1.09	0.93
PU_2_	(C_45_H_42_N_6_O_3_S_2_)_n_	42,678.09	37,998.56	~ 55	1.12	0.97
PU_3_	(C_38_H_42_N_6_O_3_S_2_)_n_	39,945.45	34,708.21	~ 58	1.15	1.06

^a^Weight-average molecular weight

^b^Number-average molecular weight

^c^Average number of repeating units

**Table 4.** TGA analyses for PU_2_ and its related PU_2_TiO_2_a-d nanocomposites

Code	IDT^a^	PDT_max_^b^	FDT^a^	Temperature (ᵒC) for various percentage decompositions*				
				T_10_	T_20_	T_30_	T_40_	T_50_
PU_2_	363.41	567.9	612.3	332.9	374.1	441.8	496.7	531.1
PU_2_TiO_2_a	364.4	571.3	614.2	338.2	374.6	444.2	489.2	521.6
PU_2_TiO_2_b	366.34	587.5	622.3	343.2	374.9	451.6	501.6	532.9
PU_2_TiO_2_c	367.48	556.3	602.3	333.1	374.2	434.1	474.1	500.6
PU_2_TiO_2_d	368.25	596.2	650.2	350.2	421.4	491.9	530.1	556.6

^a^The values were determined by TG curves at heating rate of 10°C/min

^b^The values were determined by DTG.

**Table 5.** Antimicrobial and antifungal activity of tested polyurea derivatives PU_1-3_ and its related nanocomposites PU_2_TiO_2a-d._

POLYMER	S. AUREUS	B. SUBTILIS	P. AERUGINOSA	E. COLI	C. ALBICANS
PU_1_	3	–	2	–	–
PU_2_	2	2	3	2	3
PU_3_	–	–	2	–	2
PU_2_TiO_2_a	8	5	8	6	4
PU_2_TiO_2_b	9	8	10	7	6
PU_2_TiO_2_c	11	10	9	12	8
PU_2_TiO_2_d	12	10	12	14	10

### 2.7. Molecular docking method

All docking studies were performed using the MOE program. Structural optimization of compounds 3a and 3b was performed using ChemBioDraw ultra, and their 3D structures were constructed using ChemBio3D ultra 13.0 software Molecular Modelling and Analysis, Cambridge Soft Corporation; they were then energetically minimised using MOPAC and saved as MDL Mol File (*.mol). The target crystal structures were retrieved from the Protein Data Bank (http://www.rcsb.org/pdb/). All bound water ligands and cofactors were removed from the protein, and the water molecules around the duplex were also removed before adding the hydrogen atoms. The parameters and charges were assigned with the MMFF94x force field. After alpha-site spheres were generated using the site finder module of MOE, the structural model of complexes was docked on the surface of the interior of the minor groove using the DOCK module of MOE [39–41]. All calculations were performed on an Intel(R) core (TM)i7, 3.8 GHz-based machine with MS Windows 10 as the operating system. The Dock scoring in MOE software was done utilizing the London dG scoring function and has been upgraded using two unrelated refinement methods. In addition, auto rotatable bonds were allowed; the ten best binding poses were directed and analysed to achieve the best score. To compare the docking poses to the ligand in the co-crystallised structure and to obtain the RMSD of the docking pose, the database browser was used. To rank the binding affinity of the synthesised compounds to the protein molecule, the binding-free energy and hydrogen bonds between the compounds and amino acid in the receptor were used. Evaluation of the hydrogen bonds was done by measuring the hydrogen bond length, which did not exceed 3.5 Å. In addition, RMSD of the compound position compared to the docking pose was used in ranking. Both RMSD and the mode of interaction of the native ligand within the structure of the receptor were used in the standard-docked model.

## 3. Results and discussion

Three new series of polyurea derivatives containing diarylidenecyclohexanone moiety and its related TiO_2_-doped nanocomposites with different ratios (2%, 5%, 10%, 20%) were synthesised using *in situ* polycondensation methods. The new polymers and their nanocomposites were characterised by common characterization techniques. In addition, the biological screening for all the products has been studied. Furthermore, the molecular docking studies of PU_1–3_ derivatives were also displayed against ‘5FSA’ and ‘1KZN’proteins.

### 3.1. Chemistry and characterization tools

**Figure 4.**
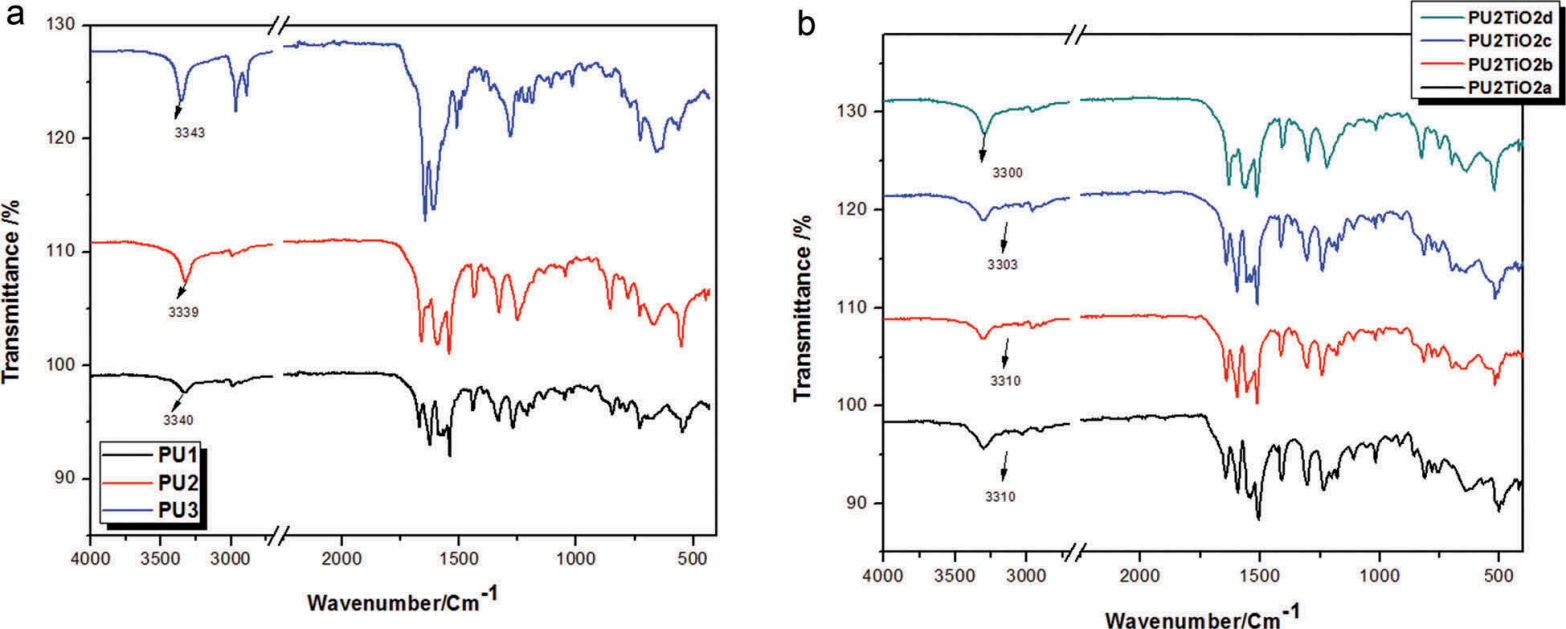
(a) FTIR spectra of polyurea derivatives PU_1_, PU_2_, and PU_3_. (b) FTIR spectra of Polyurea-TiO_2_ nanocomposites PU_2_TiO_2_a-d

M1 was synthesised using potassium hydroxide as catalysed in the mixture of 0.02 mol benzaldehyde and 0.01 mol 4-(tert-butyl)cyclohexanone in ethanol, as shown in Figure 1. The monomer structure was conﬁrmed by FTIR and ^1^H NMR as presented in the experimental section.M2 was synthesised by the interaction between M1 and chloroacetyl chloride in carbon disulfide using anhydrous aluminium chloride via the Friedel–Crafts reaction as shown in Figure 2. The monomer structure was conﬁrmed by FTIR and ^1^HNMR as presented in the experimental section. Finally, M3 was synthesised thought the interaction between M2 and thiourea in absolute ethanol then stirred under reflux and poured onto sodium acetate as shown in Figure 2. The monomer structure was conﬁrmed by FTIR and ^1^HNMR as presented in the experimental section. Subsequently, a new series of polyurea derivatives PU_1_, PU_2_, and PU_3_was synthesised using the solution polycondensation procedure [42] through the interaction of M3 with diisocyanate compounds in pyridine as presented in Figure 3. The IR spectra of the polymers showed absorption bands at 3340 cm^−1^(NH of urea derivative) and 1680 cm^−1^ (C = O of urea derivative), as shown in Figure 4(a).

The new polymers were characterised by solubility, viscosity, and GPC molecular weight determination as follows. The solubility of polyurea derivatives PU_1_, PU_2_, and PU_3_ was examined at room temperature using many solvents including formic acid, CHCl_3_, CH_2_Cl_2_, benzene, DMF, concentrated sulfuric acid A5% (w = v), and DMSO. All solutions were prepared under the same conditions, and the polyurea derivatives were soluble in concentrated H_2_SO_4_, giving a red colour. In addition, they were soluble in polar aprotic solvents like DMSO and DMF, formic acid, and CHCl_3_. However, they were only partially soluble in other solvents like methylene chloride and benzene. Table 2 presents the solubility character for the synthesized PU derivative in different solvents.

The inherent viscosities were determined for PU_1_, PU_2_, and PU_3_ in DMSO at 30°C with an Ubbelohde suspended-level viscometer. The value is defined as

$$\eta_{inh}=\;2.3\;\left[log\eta_{/}\eta_{o}\right].$$

Solution concentrations were 0.5 g/100 mL and the viscosity ratio is η/η_o_. Table 3 shows the viscosity value of PU_1_, PU_2_, and PU_3_. All polymers show viscosity values in the same range due to their very near M. Wt. values. This observation has been confirmed by the GPC measurement. Additionally, it shows that PU_1_ had a low viscosity (0.93 dl/g) among PU_1-3_. The inherent viscosity (η_inh_) values for polyurea derivatives were different for each derivative, which may result in small differences in their molecular weights.

The chromatographs have different techniques used to determine the molecular weight of polymers such as column chromatography, paper chromatography, high-performance liquid chromatography (HPLC), and GPC or by other wards size exclusion chromatography (SEC) [43–45]. These different techniques pass the solution for the tested sample through a medium that selectively absorbs the different components in the tested sample solution. GPC is extensively used for molecular weight determination. The value of the molecular weight was computed using a computer program. The value of average number, weight-average molecular weights, and polydispersity index (Mn, Mw, and PDI) of polyurea were determined and their data are presented in Table 3.

### 3.2. Polyurea-based TiO_2_nanocomposites fabrication

The same procedure was used to synthesise polyurea PU_2_ using the solution polycondensation technique by first dissolving TiO_2_ with different ratios (2%,5%,10%, and 20%) in dry pyridine and then dissolving one mole of M3 with one mole of 4,4ʹ-diphenylmethane diisocyanate compound. The pyridine polymer and nanocomposites information are presented in Table 1. The polyurea and nanocomposite structures were conﬁrmed by FTIR as presented in the experimental section. The IR spectra of PU_2_TiO_2_a–d show absorption bands at 3330 cm^−1^(NH of urea derivative) and 1660 cm^−1^(C = O of urea derivative), as shown in Figure 4(b).

The resulting polyurea derivatives and nanocomposites were characterised using XRD and TGA to determine the thermal stability of polyurea derivatives and nanocomposites and the influence of variable TiO_2_ nanoparticles concentration on their thermal stability. The morphology exhibits features associated with agglomeration and concentration of TiO_2_ nanoparticles on the polyurea surface as seen in SEM and TEM images.

**Figure 5.**
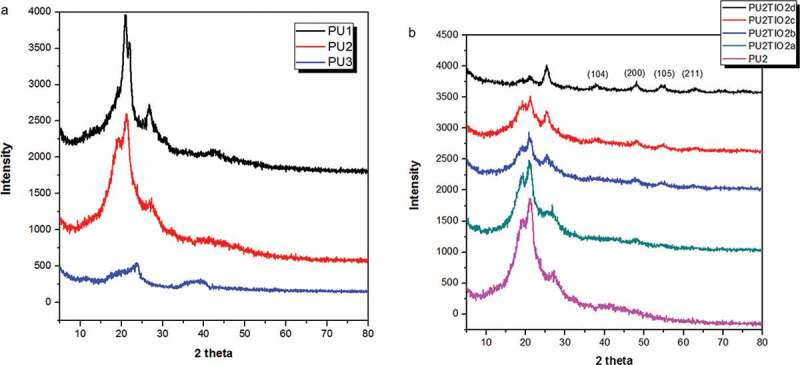
(a) X-ray diffraction patterns of PU_1_, PU_2_, PU_3._ (b) X-ray diffraction patterns of Polyurea-TiO_2_ nanocompositesPU_2_TiO_2_a-d

The fabricated nanocomposites were characterised by XRD, SEM, TEM, and TGA. First, polyurea derivatives were measured as shown in Figure 5(a). PU_1_had four characteristic peaks at 2-theta values of 20.91°, 21.94°, 26.37°, and 42.8°, and d-space values of 4.5 Å, 4.2 Å, 3.51 Å, and 2.5 Å. PU_2_hadfour characteristic peaks at 2-theta values of 19.72°, 21.18°, 26.15°, and 41.18°, and d-space values of 4.242 Å, 4.048, Å3.332 Å, and 2.11 Å. PU_3_had two characteristic peaks at 2-theta values of 19.72°and 37.42° with d-space values of 3.78 Å and 2.4 Å. PU_3_ was crystalline or semi-crystalline, possibly because of the six methylene groups, which might be the result of increasing polyurea chain flexibility in adjacent chains [46]. Additionally, the presence of the high C = C band and C = O band, which represent polar groups arranged between the adjacent polyurea chains, could have caused the extended crystallinity [47]. The XRD patterns for polyurea-based TiO_2_ nanocomposites with different percentages of TiO_2_ nanoparticles (2, 5, 10, and 20%) were measured as shown in Figure 5(b). The XRD patterns showed that polyurea with TiO_2_ nanoparticles had characteristic peaks at 2-theta values of 25.25°, 36.96°, 37.93°, 38.61°, 48.1°, 53.89°, 55.30°, 63.2°, and 69.6°, andTiO_2_ peak indices (101), (104), (200), (105), and (211) Compared to a standard card (00–002-0387), the TiO_2_ nanoparticles peaks match those of anatase. Moreover, the XRD results for these nanocomposites also indicate a gradual decrease in the PU_2_ related peaks as a result from the increase in the TiO_2_loading percent from PU_2_TiO_2_a to PU_2_TiO_2_d.

**Figure 6.**
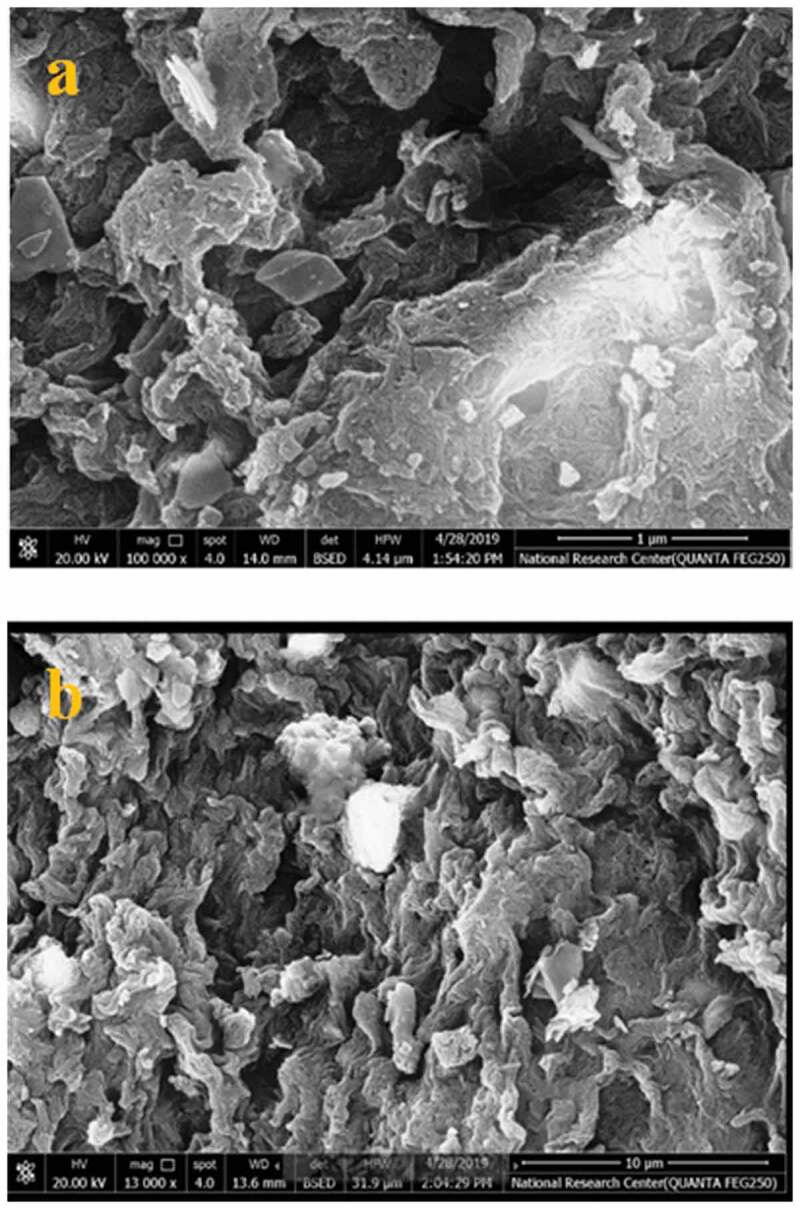
SEM images of PU_2_TiO_2_b at higher and lower magnifications, respectively: (a) X = 100,000, (b) X = 13,000

The morphology of selected samples PU_2_TiO_2_b and PU_2_TiO_2_d were examined as shown in Figure 6(a). At magniﬁcation X = 100,000, thePU_2_TiO_2_b surface was comprised of ﬁbrous structures and the addition of 5 wt. % TiO_2_ to the polymer caused agglomeration on the surface of the polymer. Additionally, in Figure 6(b), at magnification X = 13,000 in PU_2_TiO_2_d, the fibre morphology of polyurea and spherical morphology of 20 wt.% of TiO_2_ nanoparticles showed excellent homogenous size distribution of TiO_2_ nanoparticles on the polyurea surface.

**Figure 7.**
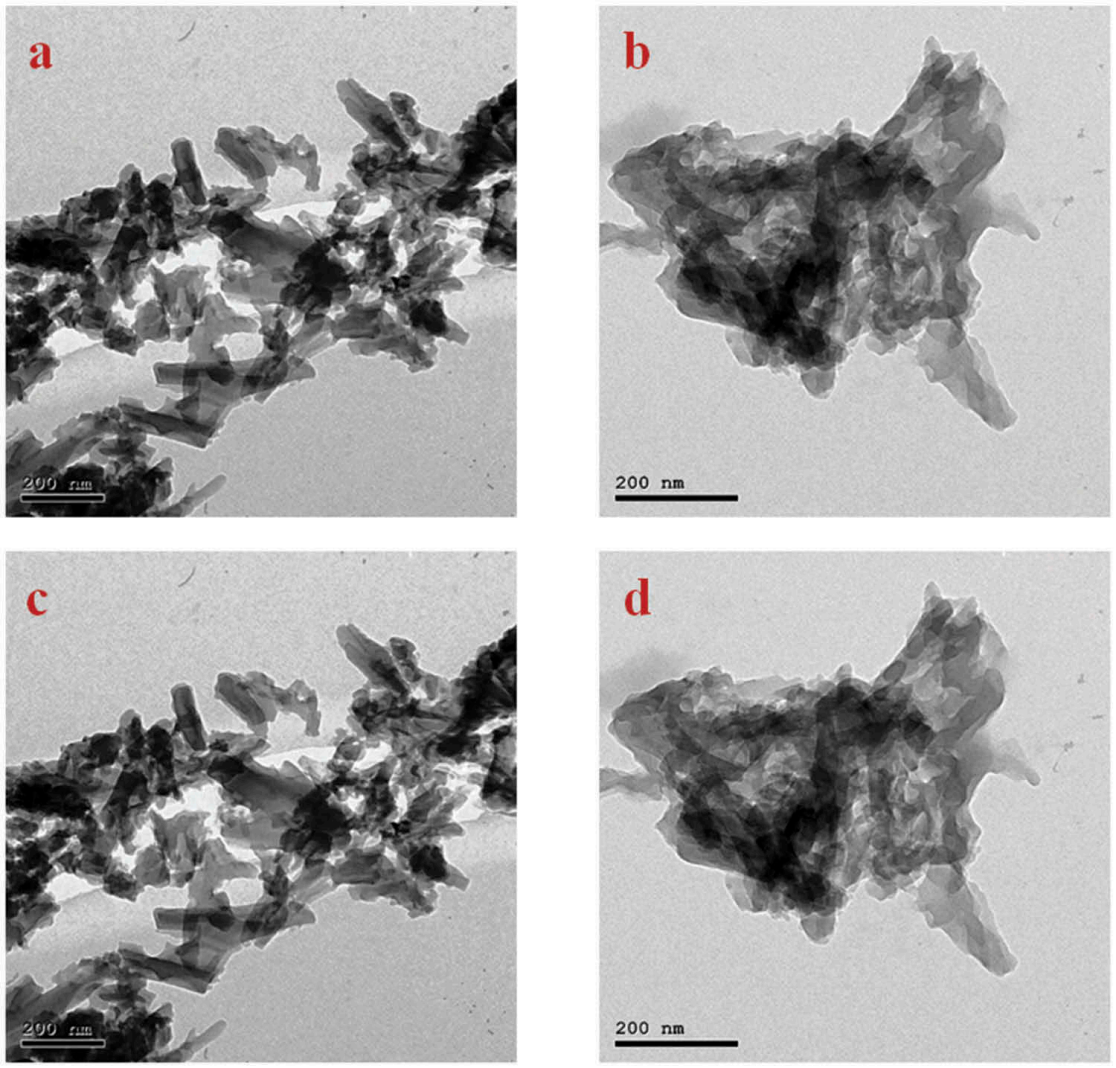
TEM images of PU_2_TiO_2_d nanocomposite

The TEM image for PUTIO_2_d (TiO_2_ nanoparticles 20%) in Figure 7 shows the spherical TiO_2_ nanoparticles dispersed in polyurea (fibre shape) with homogenous size, shape, and distribution without any agglomeration or concentration in certain areas. TiO_2_ nanoparticles are approximately 25 nm.

**Figure 8.**
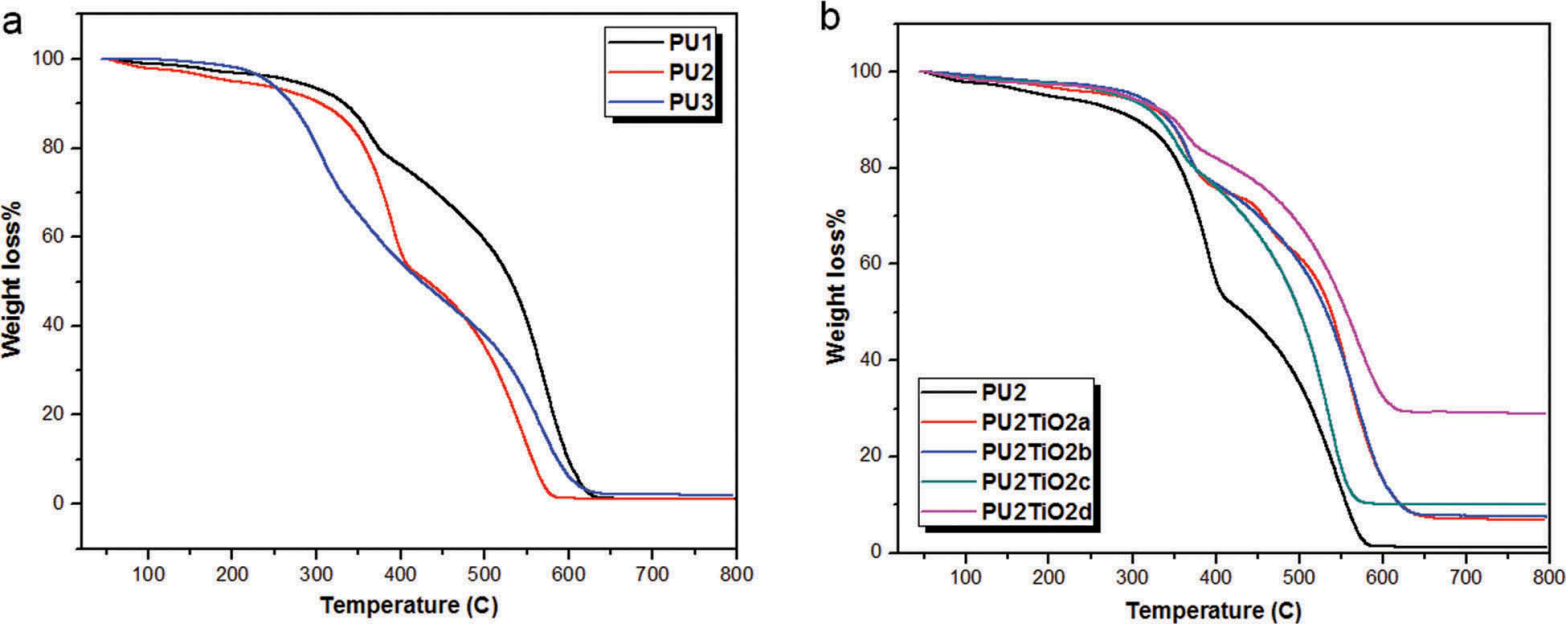
(a) TGA curves of PU_1_, PU_2_, PU_3_ in airflow at a heating rate of 10ᵒC/min. (b) TGA curves of polyurea-TiO_2_ nanocomposites PU_2_TiO_2_a-d in airflow at a heating rate of 10 ᵒ C/min

The thermographs of polyurea derivative samples are given in Figure 8(a), which shows the same decomposition curve for all samples with multi-step processes, starting with conformable removal of the (OH) group due to the removal of moisture content and/or entrapped solvents that cause weight loss; however, this step starts at room temperature and ends at approximately 160°C for PU_1_, PU_2_, and PU_3_ with mass losses 0.338, 0.245, and 0.025 mg, respectively. The thermographs also show that polyurea derivatives decompose in two stages. The first stage is the partial decomposition of polymers, which starts at 160 C° and ends at 368°C, 345°C, and 337 C° for PU_1_, PU_2_, and PU_3_, with mass losses of 2.40, 3.261, and 3.50 mg, respectively. The second stage of decomposition starts at 350°Cand ends at 612°C, 595°C, and 580°C for PU_1_, PU_2_, and PU_3_. The total mass loss of polyurea derivatives shows higher stability for PU_2_ than other polyurea derivatives with total mass loss at 800°Cof 4.44 mg while PU_1_ and PU_3_ are 16.06 and 8.063 mg, respectively. Additionally, the TGA of polyurea–TiO_2_ nanocomposite with different percentages of TiO_2_, as shown in Figure 8(b), was performed to compare the thermal stability of each nanocomposite and explain the effect of TiO_2_ nanoparticles on the thermal resistance of polyurea. The TGA curves for PU_2_ and PU2TiO2a with different percentages of TiO_2_ nanoparticles illustrate the effect of TiO_2_ nanofiller on the thermal stability of polymers as shown in Figure 8(b). The TGA data analysis shows the high thermal stability for PUTiO_2_d (20% TiO_2_ nanofiller) with total mass loss of 71% compared to PUTiO_2_a, PUTiO_2_b, and PUTiO_2_ c, which has losses of 94.66%, 94.54%, and 90.44%, respectively. The thermal properties are enhanced for all samples due to the high thermal resistance of the TiO_2_ nanofiller. The initial decomposition temperature (IDT) at which the initial degradation may occur [48,49] was found to be in the range between 300°C and 560°C. T_10_ was considered as the polymer decomposition temperature (PDT) with a range from 333°C to 350.2°C. Therefore, the data in Table 4 indicate the high thermal stability of PUTiO_2a–d_ compared to individual polyurea. PDT*_max_* represents the maximum temperature at which the decomposition process occurs [49,50]. PDT*_max_* results show that all the polymers have similar PDT*_max_* values in the range from 531°C to 560°C. The final decomposition temperature (FDT) is the temperature at which the amount of degradation that may occur is nearly completed [8,51]. The TG curves show that the FDT for all polymers is almost completed at around 600–650.2°C. By comparison, at the T_40_ and T_50_ values, the thermal stability increased with the increased percent of TiO_2_ nanoparticles.

### 3.3. Antimicrobial Evaluation

**Figure 9.**
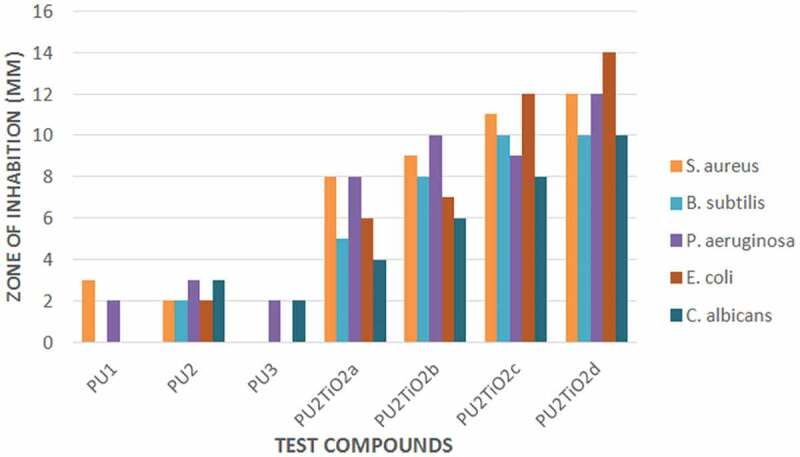
Antibacterial and antifungal activities of tested polyurea derivatives and polyurea-TiO_2_ nanocomposites

**Figure 10.**
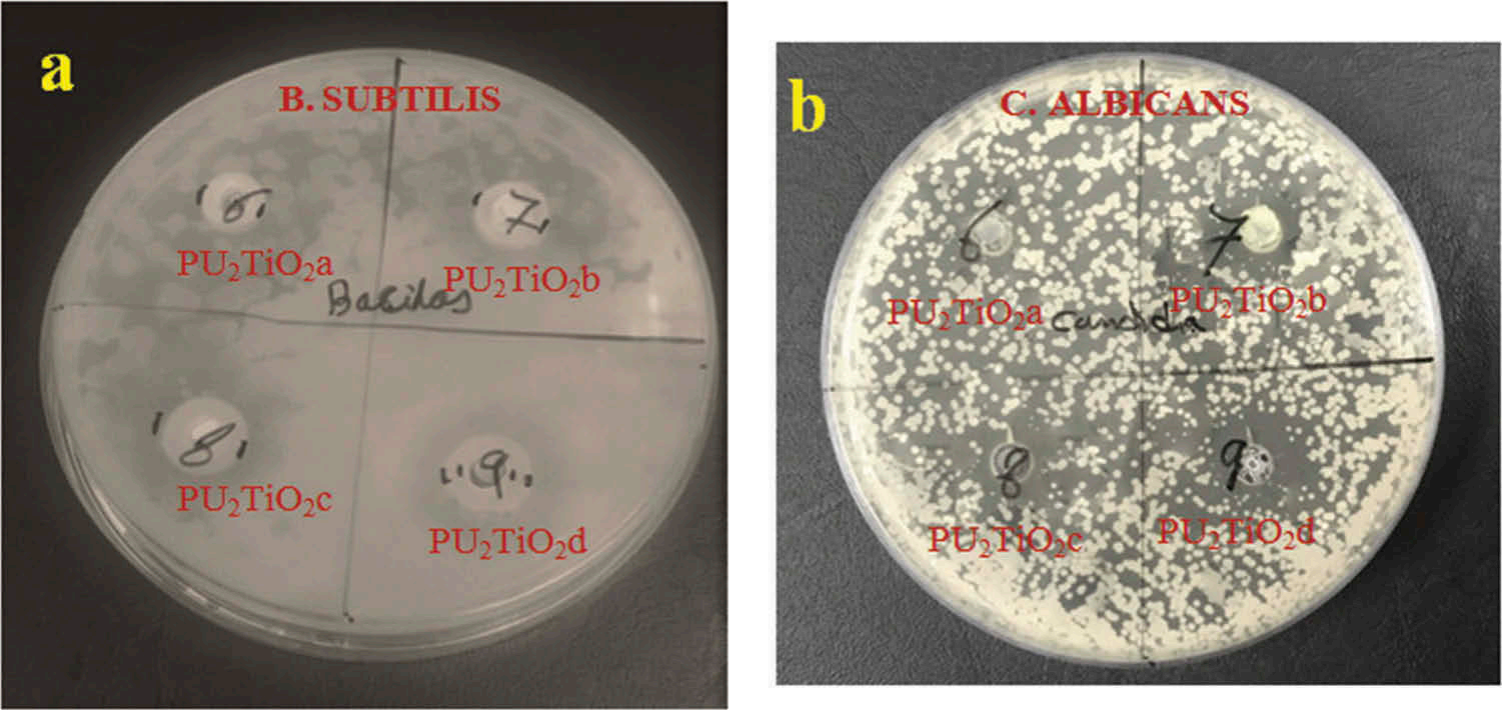
Antimicrobial activity images of tested polyurea-TiO_2_ nanocomposites against *B. subtilis*and *C. Albicans.*

The antimicrobial activity of the polyurea derivatives and TiO_2_-based nanocomposites was determined through the disk diffusion system with different gram positives of *B. subtilis and S. aureus*, and gram negatives of *E. coli* and *P. aeruginosa* bacteria as well as the fungus *C. albicans* samples. The antimicrobial activity was evaluated by the inhibition zone diameters as presented in Figures 9 and Figures 10 and Table 5. The tested polyurea derivatives showed activity against some of the microbial strains but the antibacterial activity against *B. subtilis, S. aureus, E. coli, P. aeruginosa*, and the fungus*C. albicans*was enhanced after addition TiO_2_ nanoparticles. However, in some previous studies, the addition of TiO_2_ to composites reduced bacterial attachment to the polymer surface [52–55]. But in most other cases it is reported as a good candidate which promotes the biological screening of materials [56–61]. The increase in added TiO_2_ (2%, 5%, 10%, and 20%) showed increased antibacterial activity against all the microbial strains. The maximum antibacterial activity was observed against *E. coli*, with an inhibition zone of 14 mm for PU_2_TiO_2_d; therefore, the nanocomposites were more active against E. coli than other types of bacteria, so it is clear that with the increase in TiO_2_ concentration, the zone of inhibition increased.

### 3.4. Molecular docking study

**Figure 11.**
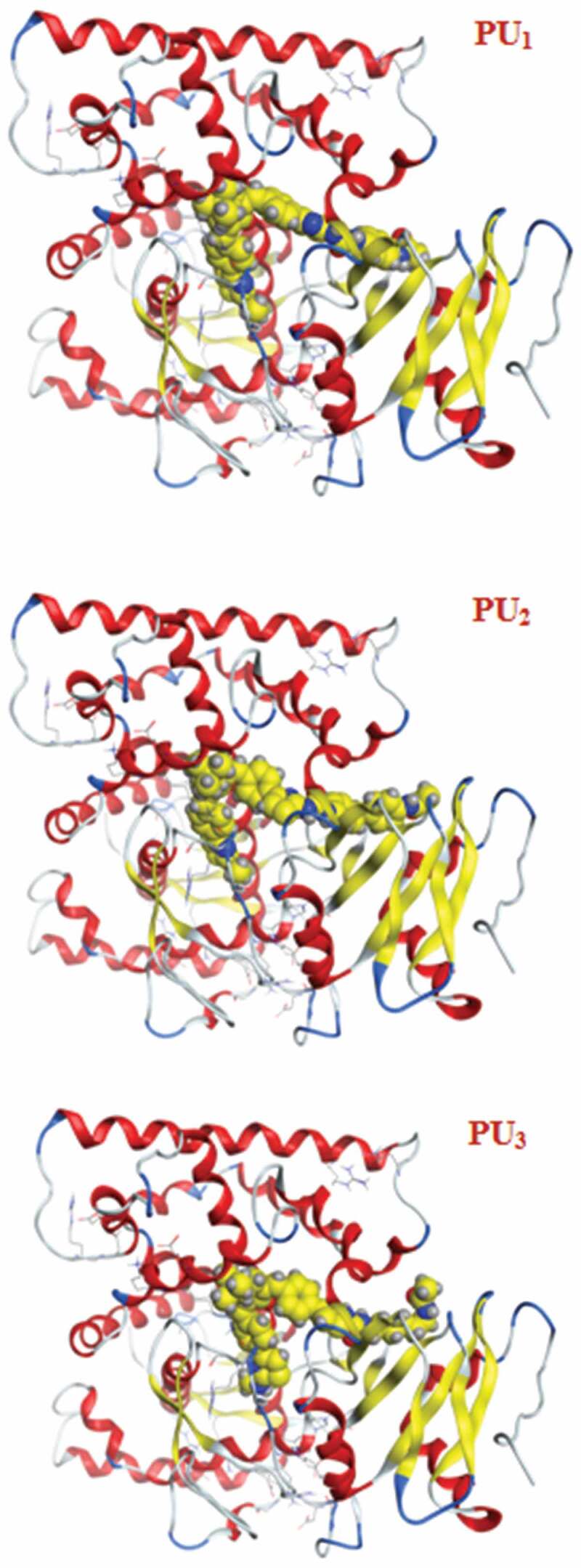
3D Docking structures of PU_1_, PU_2_, and PU_3_ with 5fsa receptor

**Figure 12.**
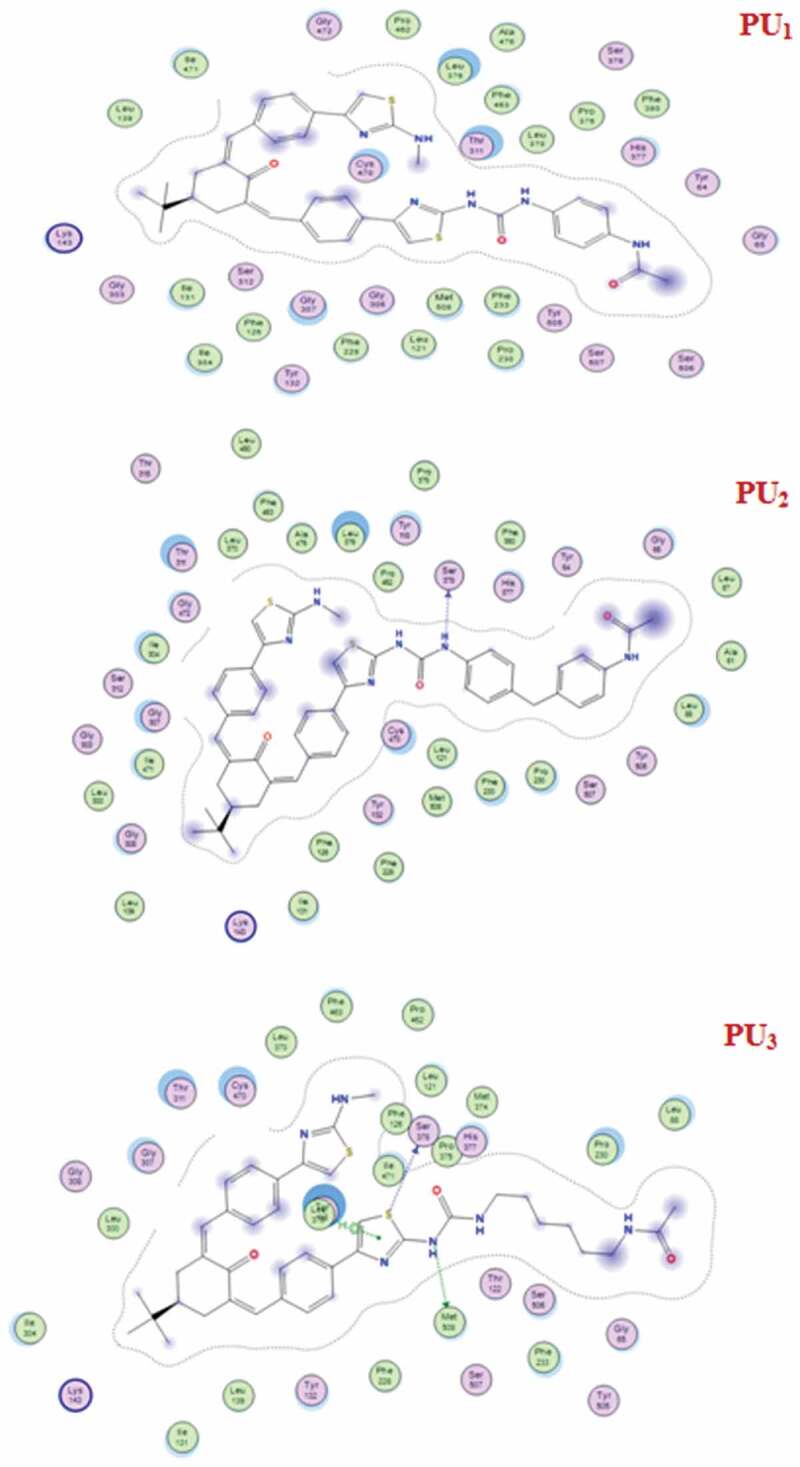
2D Docking structures and Distance between PU_1_, PU_2_, and PU_3_ with 5fsa receptor

**Figure 13.**
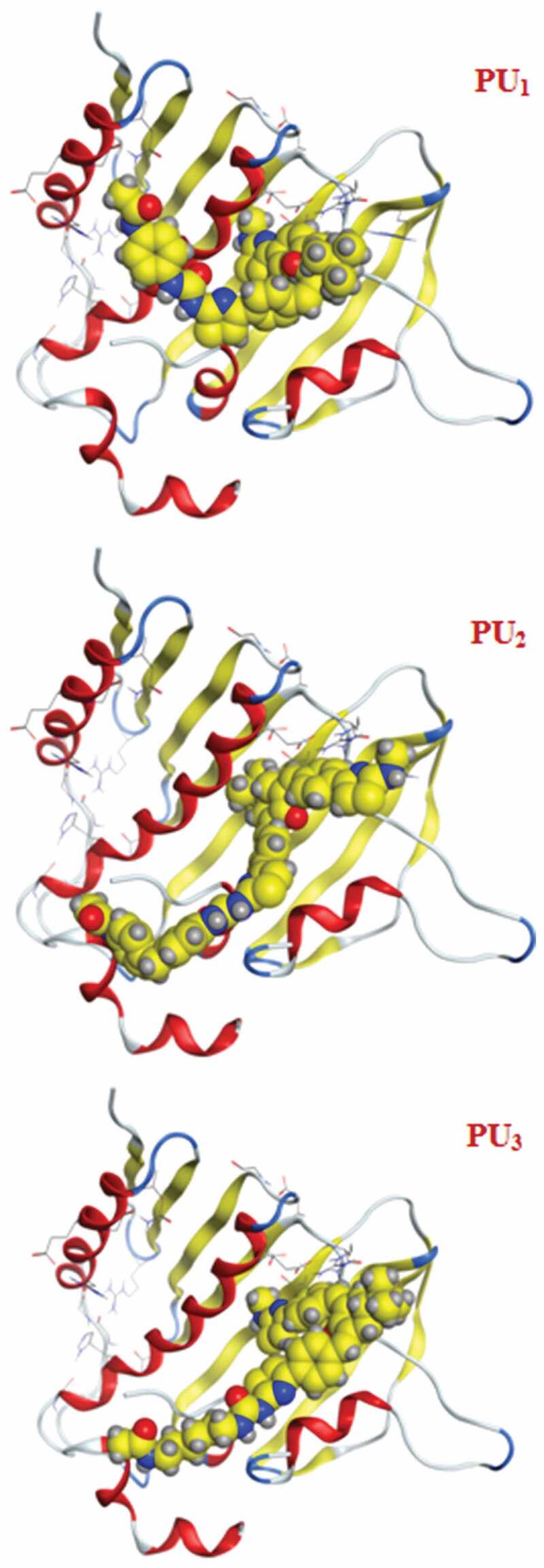
3D Docking structures of PU_1_, PU_2_, and PU_3_ with 5fsa receptor

**Figure 14.**
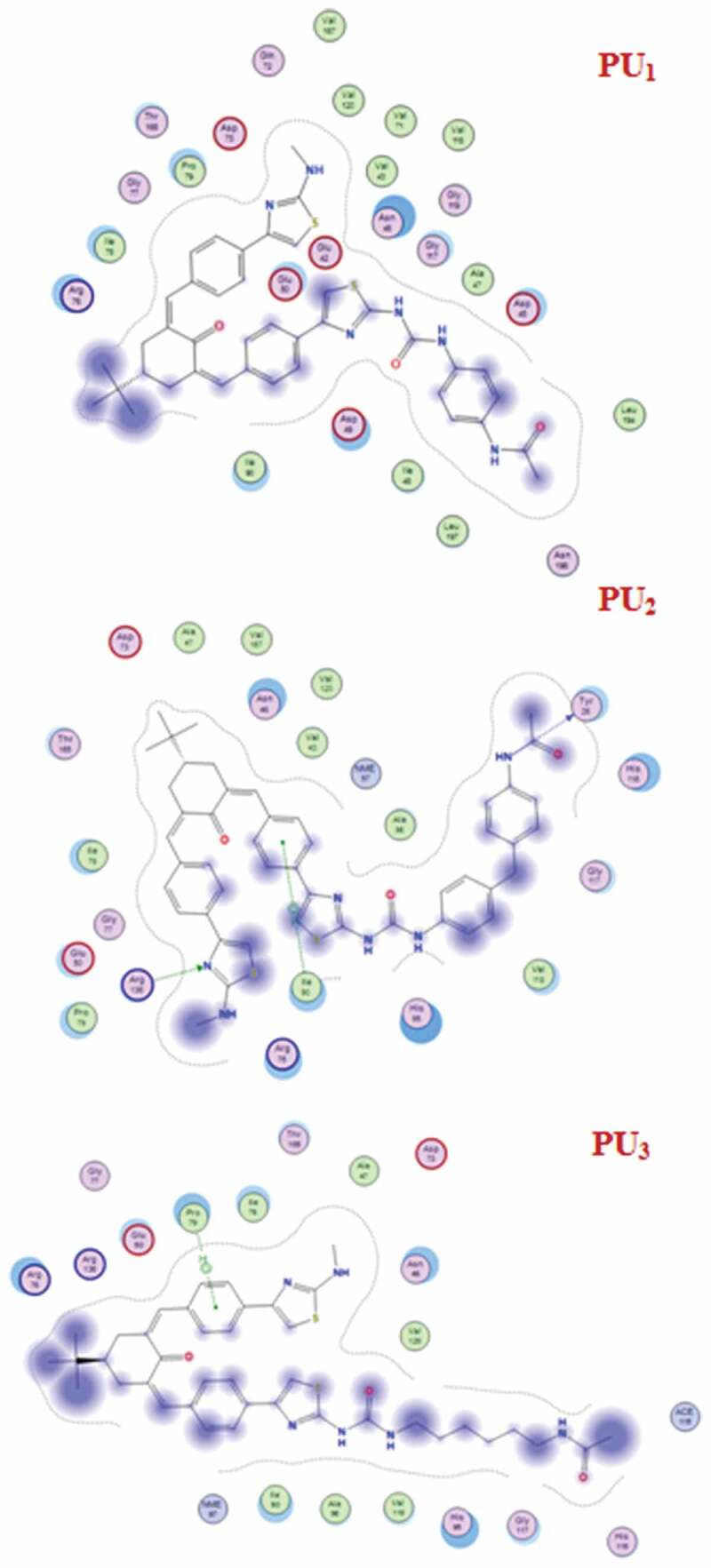
Distance and energy between PU_1_, PU_2_, and PU_3_ with 1KZN receptor

The docking studies have been applied to PU_1_, PU_2_, and PU_3_ compounds against the ‘5FSA’ protein [62,63] and ‘1KZN’ [64,65] to highlight the possible acting functional groups. 5FSA is the sterol 14-alpha demethylase (cyp51), which is a cytochrome P450 enzyme that is employed for the biosynthesis of sterols in cells and is the major target of clinical drugs in fungi [62]. 1KZN is a code for the 24 kDa gyrase fragment; DNA gyrase is a primary protein involved in replication and transcription of bacterial circular DNA [64]. Many antibacterial drugs are known to target DNA gyrase, inducing bacterial death [64]; a similar docking study was undertaken on the clinically approved drugs Gentamycin for the 1KZN protein and Fluconazole for 5FSA. The docking of 1KZN against the compounds and the antibacterial reference suggest an increase in the docking score of PU_2_over PU_1_ and PU_3_ (Table 6–9). The docking score of PU_2_ compared to PU_1_ and PU_3_ (Figures 11–13–Figures 14) can be attributed to both electronic interaction and its orientation inside the protein sites. Similarly, the docking study of 5FSA against the compounds and the antifungal reference ‘Fluconazole’ revealed similar observations. The docking scores are in good agreement with the antimicrobial effectiveness of the compounds against *E. coli* and the antifungal capacities of the compounds against *S. aureus, B. subtilis*, and *P. aeruginosa*.

**Table 6.** Docking score and energies of PU_1_, PU_2_, and PU_3_ with 5fsa receptor

mseq	S	rmsd_refine	E_conf	E_place	E_refine	h_logD	h_logP	h_logS
PU_1_	−11.90	2.39	−127.14	−102.13	−66.30	8.70	9.25	−11.71
PU_1_	−11.89	2.82	−131.02	−105.91	−63.60	8.70	9.25	−11.71
PU_1_	−11.87	1.79	−123.67	−79.06	−67.66	8.70	9.25	−11.71
PU_1_	−11.71	2.92	−134.36	−83.77	−68.96	8.70	9.25	−11.71
PU_1_	−11.54	1.83	−95.81	−47.29	−64.98	8.70	9.25	−11.71
PU_2_	−12.92	3.98	−97.54	−84.27	−72.99	10.78	11.33	−13.71
PU_2_	−12.78	3.10	−117.60	−90.28	−79.61	10.78	11.33	−13.71
PU_2_	−12.75	2.59	−109.11	−57.11	−63.60	10.78	11.33	−13.71
PU_2_	−12.49	1.92	−127.03	−87.50	−73.70	10.78	11.33	−13.71
PU_2_	−12.43	1.91	−115.05	−111.26	−65.02	10.78	11.33	−13.71
PU_3_	−12.11	2.13	−132.83	−97.56	−70.86	7.81	8.37	−10.87
PU_3_	−11.86	1.88	−119.59	−116.25	−67.15	7.81	8.37	−10.87
PU_3_	−11.80	3.56	−123.73	−84.80	−61.64	7.81	8.37	−10.87
PU_3_	−11.70	2.08	−109.67	−139.36	−52.77	7.81	8.37	−10.87
PU_3_	−11.70	1.84	−106.54	−65.27	−52.11	7.81	8.37	−10.87

PU_2_TiO_2_c PU_2_TiO_2_d

**Table 7.** Distance and energy between PU_1_, PU_2_, and PU_3_ with 5fsa receptor

Product	Ligand	Receptor	Interaction	Distance	E (kcal/mol)
PU_1_	-	-	-	-	-
PU_2_	N 71	O SER 378 (A)	H-donor	3.29	−2.1
PU_3_	S 42	O SER 378 (A)	H-donor	3.68	−0.5
	N 43	SD MET 508 (A)	H-donor	4.07	−2.7
	5-ring	CD1 LEU 376 (A)	pi-H	3.86	−1.0

**Table 8.** Docking score and energies of PU_1_, PU_2_, and PU_3_ with 1KZN receptor

mseq	S	rmsd_refine	E_conf	E_place	E_refine	h_logD	h_logP	h_logS
PU_1_	−10.12	1.52	−135.34	−102.54	−60.59	8.70	9.25	−11.71
PU_1_	−9.61	2.42	−142.01	−105.79	−63.16	8.70	9.25	−11.71
PU_1_	−9.46	2.50	−140.00	−82.31	−59.95	8.70	9.25	−11.71
PU_1_	−9.43	3.01	−139.54	−78.94	−60.70	8.70	9.25	−11.71
PU_1_	−9.19	2.02	−142.39	−89.25	−59.09	8.70	9.25	−11.71
PU_2_	−10.38	2.21	−119.74	−70.35	−58.54	10.78	11.33	−13.71
PU_2_	−9.94	2.29	−127.41	−46.42	−63.84	10.78	11.33	−13.71
PU_2_	−9.82	2.14	−133.69	−73.17	−68.89	10.78	11.33	−13.71
PU_2_	−8.81	2.43	−126.65	−36.69	−51.48	10.78	11.33	−13.71
PU_2_	−8.69	1.94	−133.67	−87.72	−50.66	10.78	11.33	−13.71
PU_3_	−8.24	2.23	−134.24	−79.43	−47.01	7.81	8.37	−10.87
PU_3_	−8.17	3.93	−139.43	−60.48	−47.30	7.81	8.37	−10.87
PU_3_	−8.17	1.88	−133.60	−70.42	−49.44	7.81	8.37	−10.87
PU_3_	−8.06	2.49	−136.33	−55.69	−46.41	7.81	8.37	−10.87
PU_3_	−7.99	5.07	−138.08	−76.02	−54.55	7.81	8.37	−10.87

**Table 9.** 2D Docking structures and Distance between PU_1_, PU_2_, and PU_3_ with 1KZN receptor

Product	Ligand	Receptor	Interaction	Distance	E (kcal/mol)
PU_1_	–	–	–	–	–
PU_2_	N 97	O TYR 26 (A)	H-donor	3.01	−4.1
	N 48	NH1 ARG 136	(A) H-acceptor	3.09	−0.9
	6-ring	CD1 ILE 90 (A)	pi-H	4.24	−0.8
PU_3_	6-ring	CD PRO 79 (A)	pi-H	4.55	−0.8

## 4. Conclusions

A series of polyurea derivatives and polyurea–TiO_2_ nanocomposites were successfully synthesised in pyridine using the polycondensation technique. The structure was confirmed by FTIR, and characterised using XRD, TGA, and SEM. TGA showed a high thermal stability for polyurea–TiO_2_ nanocomposites with the increase in TiO_2_nanoparticles. The synthesised TiO_2_ nanocomposites emerged as good antimicrobial agents. TEM images for PU2TiO_2_d showed the spherical TiO_2_ nanoparticles dispersed into polyurea (fibre shape), also SEM images for PU_2_TiO_2_b and PU_2_TiO_2_d showed a ﬁbrous polymer structure with spherical morphology for the TiO_2_. The polyurea derivatives showed slight antibacterial activity and after adding TiO_2_ nanoparticles showed activity against all the microbial strains; the activity increased with the increase in TiO_2_ present.

## Supplementary Material

Supplemental Material

## References

[1] Chantarasiri N, Chulamanee C, Mananunsap T, et al. Thermally stable metal-containing polyureas from hexadentate Schiff base metal complexes and diisocyanates. Polym Degrad Stab. 2004;86(3):505–513.

[2] Mallakpour S, Raheno H. Synthesis and characterization of new polyureas based on 4-(4′-aminophenyl) urazole and various diisocyanates. J Appl Polym Sci. 2003;89(10):2692–2700.

[3] Lin JK, Yuki Y, Kunisada H, et al. Synthesis and characterization of new aromatic polyamides, polyimides, and polyureas containing 1, 3, 5‐triazine rings. J Appl Polym Sci. 1990;40(11-12:2113–2122.

[4] Melissaris AP, Mikroyannidis JA. Phosphorus-containing crosslinkable polymers for fire- and heat-resistant applications. Eur Polym J. 1989;25(3):275–280.

[5] Miyamoto M, Takashima Y, Kimura Y. Preparation of novel thermally stable polyurea by the cationic ring-opening isomerization polymerization of polycyclic pseudourea. Macromolecules. 1998;31(20):6822–6827.

[6] Matsuda H, Takechi S. Syntheses and properties of polyureas from divalent metal salts of p‐aminobenzoic acid, diamine, and diisocyanate. J Polym Sci A Polym Chem. 1990;28(7):1895–1908.

[7] Qiu W, Zeng W, Zhang X, et al. Preparation and characterization of polyureas from divalent metal (Ba, Sr, Pb, Zn) salts of sulfanilic acid. J Appl Polym Sci. 1993;49(3):405–415.

[8] Aly K, Hussein M. Synthesis, characterization and corrosion inhibitive properties of new thiazole based polyamides containing diarylidenecyclohexanone moiety. Chin J Polym Sci. 2015;33(1):1–13.

[9] Lagrenee M, Mernari B, Bouanis M, et al. Study of the mechanism and inhibiting efficiency of 3, 5-bis (4-methylthiophenyl)-4H-1, 2, 4-triazole on mild steel corrosion in acidic media. Corros Sci. 2002;44(3):573–588.

[10] Bentiss F, Lagrenee M, Traisnel M, et al. The corrosion inhibition of mild steel in acidic media by a new triazole derivative. Corros Sci. 1999;41(4):789–803.

[11] Lukovits I, Kalman E, Palinkas G. Nonlinear group-contribution models of corrosion inhibition. Corrosion. 1995;51(3):201–205.

[12] Yao B, Wang G, Ye J, et al. Corrosion inhibition of carbon steel by polyaniline nanofibers. Mater Lett. 2008;62(12–13):1775–1778.

[13] Sathiyanarayanan S, Jeyaprabha C, Venkatachari G. Influence of metal cations on the inhibitive effect of polyaniline for iron in 0.5 M H2SO4. Mater Chem Phys. 2008;107(2–3):350–355.

[14] Kilmartin PA, Trier L, Wright GA. Corrosion inhibition of polyaniline and poly (o-methoxyaniline) on stainless steels. Synth Met. 2002;131(1–3):99–109.

[15] Aly KI, Abbady MA, Mahgoub SA, et al. New Polymer Synthesis Part 14. Synthesis and properties of some new polyketo-amine polymers containing cycloalkanone moieties in the main chain. J Polym Int. 2002;51(2):125–133.

[16] Abbady MA, Aly KI, Mahgoub SA, et al. New polymer synthesis part 15. Synthesis and characterization of new polyketoamine polymers containing ether and thioether linkage in the main chain. J Polym Int. 2005;54(11):1512–1523.

[17] Hussein MA, Abdel-Rahman MA, Geies AA. New heteroaromatic polyazomethines containing naphthyridine moieties: synthesis, characterization, and biological screening. J Appl Polym Sci. 2012;126(1):2–12.

[18] Aly KI, Abbady MA, Mahgoub SA, et al. New polymer syntheses 44. Synthesis, characterization and corrosion inhibition behavior of new polyurea derivatives based on diaryl ether in the polymers backbone. J Appl Polym Sci. 2009;112(2):620–628.

[19] Aly KI, Abbady MA, Mahgoub SA, et al. Liquid crystalline polymers IX main chain thermotropic poly (azomethine – ether)scontaining thiazole moiety linked with polymethylene spacers. J Express Polym Lett. 2007;1(4):197–207.

[20] Aly KI, Abdel Rahman MA, Hussein MA. Synthesis and characterization of new polyamides of diarylidenecycloalkanone moieties containing Azo groups in the polymers main chain. Int J Polym Mater. 2010;59(8):553–569.

[21] Aly KI, Hussein MA. New polymer syntheses part 45. Corrosion inhibition behavior of novel polyurea derivatives based on diarylidenecycloalkanone moieties in the polymers backbone. J Polym Res. 2010;17(5):607–620.

[22] Hussein MA, Asiri AA, Aly KI. New polyamides and polyoxazoles containing diphenyl ether in the polymers backbone. Int J Polym Mater. 2012;61(2):154–175.

[23] Hussein MA, Asiri AM. Organometallic ferrocene- and phosphorus-containing polymers: synthesis and characterization. Des Monomers Polym. 2012;15(3):207–251.

[24] Hussein MA, Marwany H, Alamry KA, et al. Surface selectivity competition of newly synthesized polyarylidene(keto-amine)s polymers toward different metal ions. J Appl Polym Sci. 2014;131(19):40873 (1–10).

[25] Abu-Zied BM, Hussein MA, Asiri AM. Development and characterization of the composites based on mesoporous MCM-41 and polyethylene glycol and their properties. Composites. 2014;58:185–192.

[26] Hussein MA, Abu-Zied BM, Asiri AM. Preparation, characterization, and electrical properties of ZSM-5/PEG composite particles. Polym Composites. 2014;35(6):1160–1168.

[27] Gabal MA, Hussein MA, Hermas A-EA, et al. Electrical conductivity of polyaniline-Mn_0.8_Zn_0.2_Fe_2_O_4_ nano-composites. Int J Electrochem Sci. 2016;11:4526–4538.

[28] Alenazi NA, Hussein MA, Alamry KA, et al. Modified polyether-sulfone membrane: A mini review. Des Monomers Polym. 2017;20(1):532–546. 29491825 10.1080/15685551.2017.1398208 PMC5812116

[29] Rahman MM, Hussein MA, Alamry KA, et al. Polyaniline/graphene/carbon nanotubes nanocomposites for sensing environmentally hazardous 4-aminophenol. Nano-Struct Nano-Objects. 2018;15:63–74.

[30] Hussein MA, El-Shishtawy RM, Obaid AY. The impact of graphene nano-plates on the behavior of novel conducting polyazomethine nanocomposites. RSC Adv. 2017;7:9998–10008.

[31] Hussein MA, El-Shishtawy RM, Obaid AY, et al. Influence of single-walled carbon nanotubes on the performance of poly(azomethine-ether) composite materials. Polym-Plast Technol Eng. 2018;57(11):1150–1163.

[32] Patel JP, Xiang ZG, Hsu SL, et al. Path to achieving molecular dispersion in a dense reactive mixture. J Polym Sci B Polym Phys. 2015;53:1519–1526.

[33] Patel JP, Deshmukh S, Zhao C, et al. An analysis of the role of nonreactive plasticizers in the cross-linking reactions of a rigid resin. J Polym Sci B Polym Phys. 2017;55:206–213.

[34] Patel JP, Zhao CX, Deshmukh S, et al. An analysis of the role of reactive plasticizers in the cross-linking reactions of a rigid resin. Polymer. 2016;107:12–18.

[35] Patel JP, Xiang ZG, Hsu SL, et al. Characterization of the cross-linking reaction in high performance adhesives. Int J Adhes Adhes. 2017;78:256–262.

[36] Patel JP, Hsu SL. Development of low field NMR technique for analyzing segmental mobility of cross-linked polymers. J Polym Sci B Polym Phys. 2018;56:639–642.

[37] Aly KI, Al-Muaikel NS, Abdel-Rahman MA, et al. Liquid crystalline polymers XVI. Thermotropic liquid crystalline copoly (arylidene-ether)/TiO_2_ nanocomposites: synthesis, characterisation and applications. Liq Cryst. 2019;46(11):1734–1746.

[38] Ge B, Wang F, Sjölund-Karlsson M, et al. Antimicrobial resistance in campylobacter: susceptibility testing methods and resistance trends. J Microbiol Methods. 2013;95(1):57–67. 23827324 10.1016/j.mimet.2013.06.021

[39] Abdellattif MH, Hussien MA, Alzahrani E. New approaches of 4-aryl-2-hydrazinothiazole derivatives synthesis, molecular docking and biological evaluations. IJPSR. 2018;9:5060–5078.

[40] Al-Khathami ND, Al-Rashdi KS,  Babgi BA, et al. Spectroscopic and biological properties of platinum complexes derived from 2-pyridyl Schiff bases. J. Saudi Chem. Soc. 2019;  23: 903–915.

[41] Mashat KH, Babgi BA, Hussien MA, et al. Synthesis, structures, DNA-binding and anticancer activities of some copper(I)-phosphine complexes. Polyhedron. 2019;158:164–172.

[42] Al-Muaikel NS, Aly KI, Hussein MA. Synthesis, characterization and antimicrobial properties of new poly (ether-ketone) s and copoly (ether-ketone)s containing diarylidenecycloalkanone moieties in the main chain. J Appl Polym Sci. 2008;108(5):3138–3147.

[43] Baik YS, Cheong WJ. Determination of molecular weight distribution and average molecular weights of oligosaccharides by HPLC with a common C18 phase and a mobile phase with high water content. Bull Korean Chem Soc. 2007;28(5):847–850.

[44] García-Lopera R, Codoñer A, Bañó MC, et al. Size-exclusion chromatographic determination of polymer molar mass averages using a fractal calibration. J Chromatogr Sci. 2005;43:1–9. 10.1093/chromsci/43.5.226 15975240

[45] JANCO M, SYKORA D, SVEC F, et al. Rapid determination of molecular parameters of synthetic polymers by precipitation/redissolution high-performance liquid chromatography using “molded” monolithic column. J Polym Sci. 2000;38:2767–2778.

[46] C H L, Chang TC. Studies on thermotropic liquid crystalline polymers—part II. Synthesis and properties of poly (azomethine-ether). Eur Polym J. 1991;27(1):35–39.

[47] Mandelkern L. Crystallization of polymers. New York: McGraw-Hill; 1964.

[48] Rahman MM, Hussein MA, Aly KI, et al. Thermally stable hybrid polyarylidene(azomethine-ether)s polymers (paap): an ultrasensitive arsenic(III) sensor approach. Des Monomers Polym. 2018;21(1):82–98. 29844770 10.1080/15685551.2018.1471793 PMC5965036

[49] Hussein MA. The role of mixed graphene/carbon nanotubes on the coating performance of G/CNTs/epoxy resin nanocomposites. Int J Electrochem Sci. 2016;11:7644–7659.

[50] Hussein MA, Abu-Zied BM, Asiri AM. Fabrication of EPYR/GNP/MWCNT carbon-based composite materials for promoted epoxy coating performance. RSC Adv. 2018;8:23555–23566. 10.1039/c8ra03109f PMC9081781 35540285

[51] Hussein MA. Eco-friendly polythiophene (keto-amine) s based on cyclopentanone moiety for environmental remediation. J Polym Environ. 2018;26(3):1194–1205.

[52] Høiby N, Ciofu O, Johansen HK, et al. The clinical impact of bacterial biofilms. Int J Oral Sci. 2011;3(2):55–65. 21485309 10.4248/IJOS11026 PMC3469878

[53] Hengzhuang W, Wu H, Ciofu O, et al. Pharmacokinetics/pharmacodynamics of colistin and imipenem on mucoid and nonmucoid Pseudomonas aeruginosa biofilms. Antimicrob Agents Chemother. 2011;55:4469–4474. 21670181 10.1128/AAC.00126-11 PMC3165294

[54] Hengzhuang W, Wu H, Ciofu O, et al. In vivo pharmacokinetics/pharmacodynamics of colistin and imipenem in pseudomonas aeruginosa biofilm infection. Antimicrob Agents Chemother. 2012;56:2683–2690. 22354300 10.1128/AAC.06486-11 PMC3346607

[55] Høiby N, Bjarnsholt T, Givskov M, et al. Antibiotic resistance of bacterial biofilms. Int J Antimicrob Agents. 2010;35:322–332. 20149602 10.1016/j.ijantimicag.2009.12.011

[56] Rajh T, Dimitrijevic NM, Bissonnette M, et al. Titanium dioxide in the service of the biomedical revolution. Chem Rev. 2014;114(19):10177–10216. 25171650 10.1021/cr500029g

[57] Zhiyuan L, Shuili Y, Heedeung P, et al. Impact of titanium dioxide nanoparticles on the bacterial communities of biological activated carbon filter intended for drinking water treatment. Environ Sci Pollut Res. 2016;23:15574–15583. 10.1007/s11356-016-6742-x 27126871

[58] Ahmadi R, Tanomand A, Kazeminava F, et al. Fabrication and characterization of a titanium dioxide (TiO2) nanoparticles reinforced bio-nanocomposite containing *Miswak* (*Salvadora persica* L.) extract – the antimicrobial, thermo-physical and barrier properties. Int J Nanomedicine. 2019;14:3439–3454. 31190802 10.2147/IJN.S201626 PMC6522844

[59] Zafar N, Uzair B, Niazi MBK, et al. Fabrication & characterization of chitosan coated biologically synthesized TiO_2_ nanoparticles against PDR *E. coli* of veterinary origin. Adv Polym Technol. 2020;8456024:1–13.

[60] Abbas WA, Abdullah IH, Ali BA, et al. Recent advances in the use of TiO_2_ nanotube powder in biological, environmental, and energy applications. Nanoscale Adv. 2019;1(8):2801–2816. 10.1039/c9na00339h PMC9418402 36133585

[61] Mallakpour S, Zeraatpisheh F, Sabzalian MR. Sonochemical-assisted fabrication of biologically active chiral poly(ester-imide)/TiO2bionanocomposites derived from L-methionine and L-tyrosine amino acids. Express Polym Lett. 2011;5(9):825–837.

[62] Kaur H, Gahlawat S, Singh J, et al. Molecular docking study of active diazenyl scaffolds as inhibitors of essential targets towards antimicrobial drug discovery. Curr Drug Targets. 2019;20(15):1587–1602. 31215386 10.2174/1389450120666190618122359

[63] Hargrove TY, Friggeri L, Wawrzak Z, et al. Structural analyses of candida albicans sterol 14α-demethylase complexed with azole drugs address the molecular basis of azole-mediated inhibition of fungal sterol biosynthesis. J Biol Chem. 2017;292:6728–6743. 28258218 10.1074/jbc.M117.778308 PMC5399120

[64] Gullapelli K, Brahmeshwari G, Ravichander M, et al. Synthesis, antibacterial and molecular docking studies of new benzimidazole derivatives. Egypt J Basic Appl Sci. 2017;4(4):303–309.

[65] Lafitte D, Lamour V, Tsvetkov PO, et al. DNA gyrase interaction with coumarin-based inhibitors: the role of the hydroxybenzoate isopentenyl moiety and the 5‘-methyl group of the noviose. Biochemistry. 2002;41:7217–7223. 12044152 10.1021/bi0159837

