# Supplementary material for: Novel biologically active polyurea derivatives and its TiO2-doped nanocomposites
Source: Des Monomers Polym. 2020 May 25;23(1):59–74. doi: 10.1080/15685551.2020.1767490 (PMC7448906; doi:10.1080/15685551.2020.1767490)
Supplement: Supplemental Material [file TDMP_A_1767490_SM1022.docx]

**Supplementary information file for**

**Novel Biologically Active Polyurea Derivatives and its TiO_2_-Doped Nanocomposites**

Mahmoud A. Hussein^1,2*^ Khalid A. Alamry^1^, Samar J Almehmadi^1^, M.A. Elfaky^3^, H. Džudžević-Čančar^4^, Abdullah M. Asiri^1,5^, Mostafa A. Hussien^1,6^

^1^ Department of Chemistry, Faculty of Science, King Abdulaziz University, Jeddah 21589, Saudi Arabia

^2^ Polymer Chemistry Lab., Chemistry Department, Faculty of Science, Assiut University, Assiut, Egypt

^3^Fcaulty of Pharmacy, Natural Products and Alternative Medicine Department, King Abdulaziz University, Jeddah 21589, Saudi Arabia

^4^ Department of Natural Science in Pharmacy, Faculty of Pharmacy, University of Sarajevo, ZmajaodBosne 8, 71 000 Sarajevo, Bosnia-Herzegovina

^5^Center of Excellence for Advanced Materials Research, King Abdulaziz University, Jeddah 21589, Saudi Arabia

^6^Department of Chemistry, Faculty of Science, Port Said University, Port Said, 42521, Egypt

* **Corresponding author:** Mahmoud A. Hussein (mahussein74@yahoo.com; maabdo@kau.edu.sa; mahmali@aun.edu.sa).

**Contents**

| Figure S1. | FTIR spectrum of 2,6-dibenzylidene4-tert-butylcyclohexanone. | S3 |
| --- | --- | --- |
| Figure S2. | FTIR spectrum of bis (4-chloroacetylbenzylidene)-4-(tert-butyl) cyclohexanone. | S4 |
| Figure S3. | FTIR spectrum of bis (2-aminothiazol-4-ylbenzylidene)-4-(tert-butyl) cyclohexanone. | S5 |
| Figure S4. | ^1^H-NMR spectrum of 2,6-dibenzylidene4-tert-butylcyclohexanone . | S6 |
| Figure S5. | ^1^H-NMR spectrum of bis (4-chloroacetylbenzylidene)-4-(tert-butyl) cyclohexanone. | S7 |
| Figure S6. | ^1^H-NMR spectrum of bis (2-aminothiazol-4-ylbenzylidene)-4-(tert-butyl) cyclohexanone. | S8 |
| Figure S7. | ^1^H-NMR spectrum of bis (2-aminothiazol-4-ylbenzylidene)-4-(tert-butyl) cyclohexanone in the presence of D_2_O. | S9 |
| Figure S8. | ^13^C-NMR spectrum of 2,6-dibenzylidene4-tert-butylcyclohexanone. | S10 |
| Figure S9. | ^13^C-NMR spectrum of bis (4-chloroacetylbenzylidene)-4-(tert-butyl) cyclohexanone. | S11 |
| Figure S10. | ^13^C-NMR spectrum of bis (2-aminothiazol-4-ylbenzylidene)-4-(tert-butyl) cyclohexanone. | S12 |

Figure S1

Figure S2

Figure S3


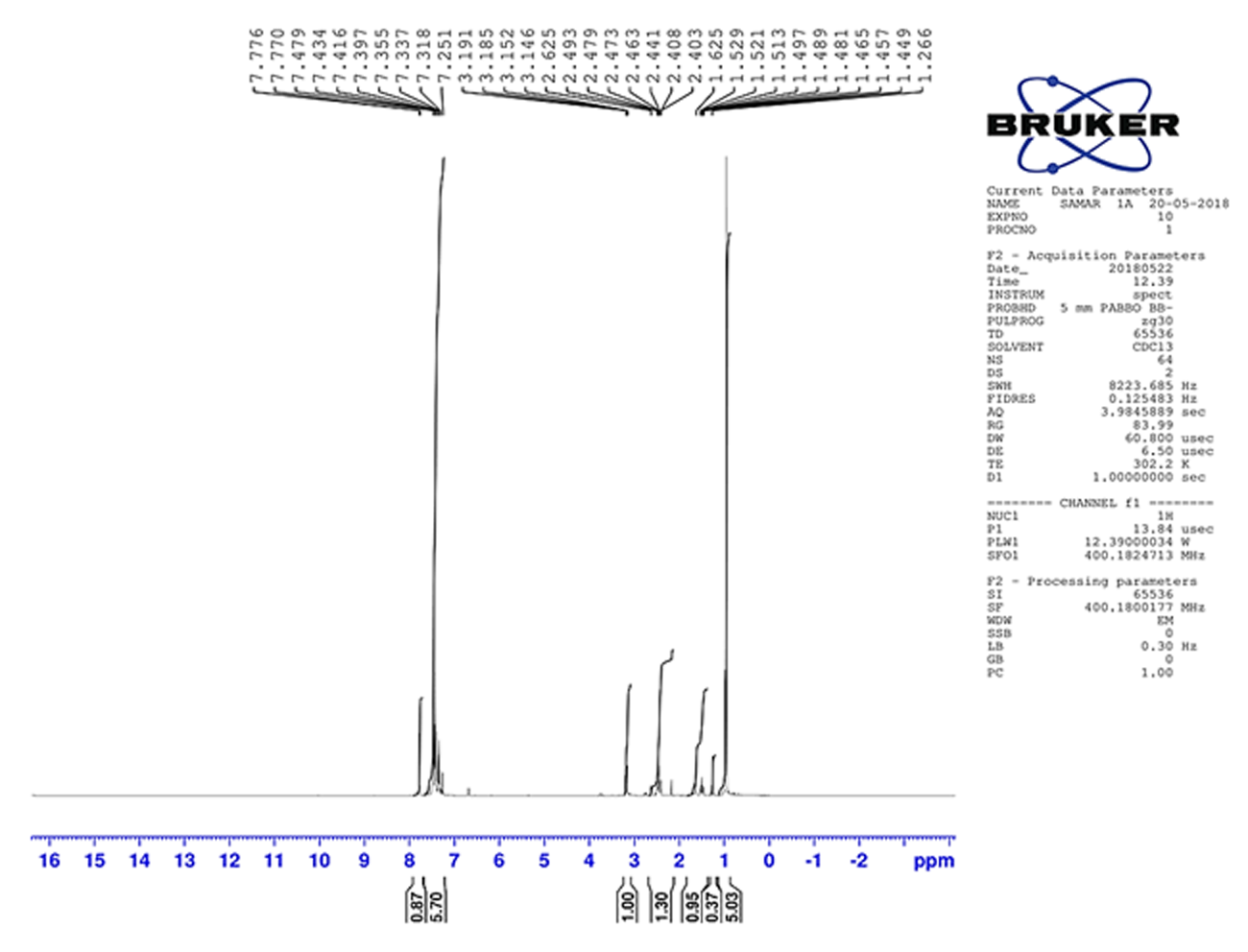


Figure S4

Figure S5

Figure S6

Figure S7


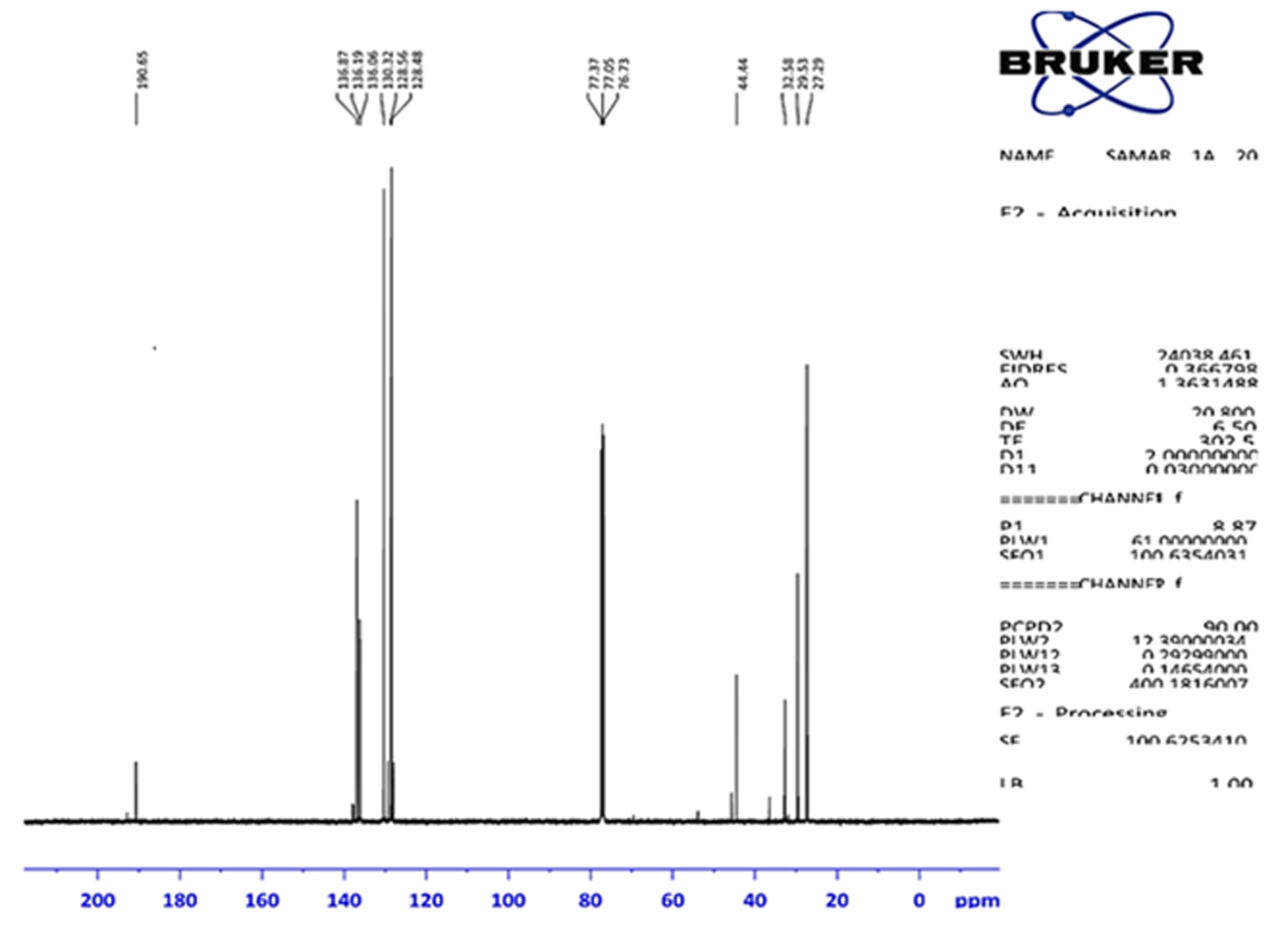


Figure S8

Figure S9

Figure S10
